# Supplementary figures and images for: Thrips as the Transmission Bottleneck for Mixed Infection of Two Orthotospoviruses
Source: Plants (Basel). 2020 Apr 15;9(4):509. doi: 10.3390/plants9040509 (PMC7238027; doi:10.3390/plants9040509)

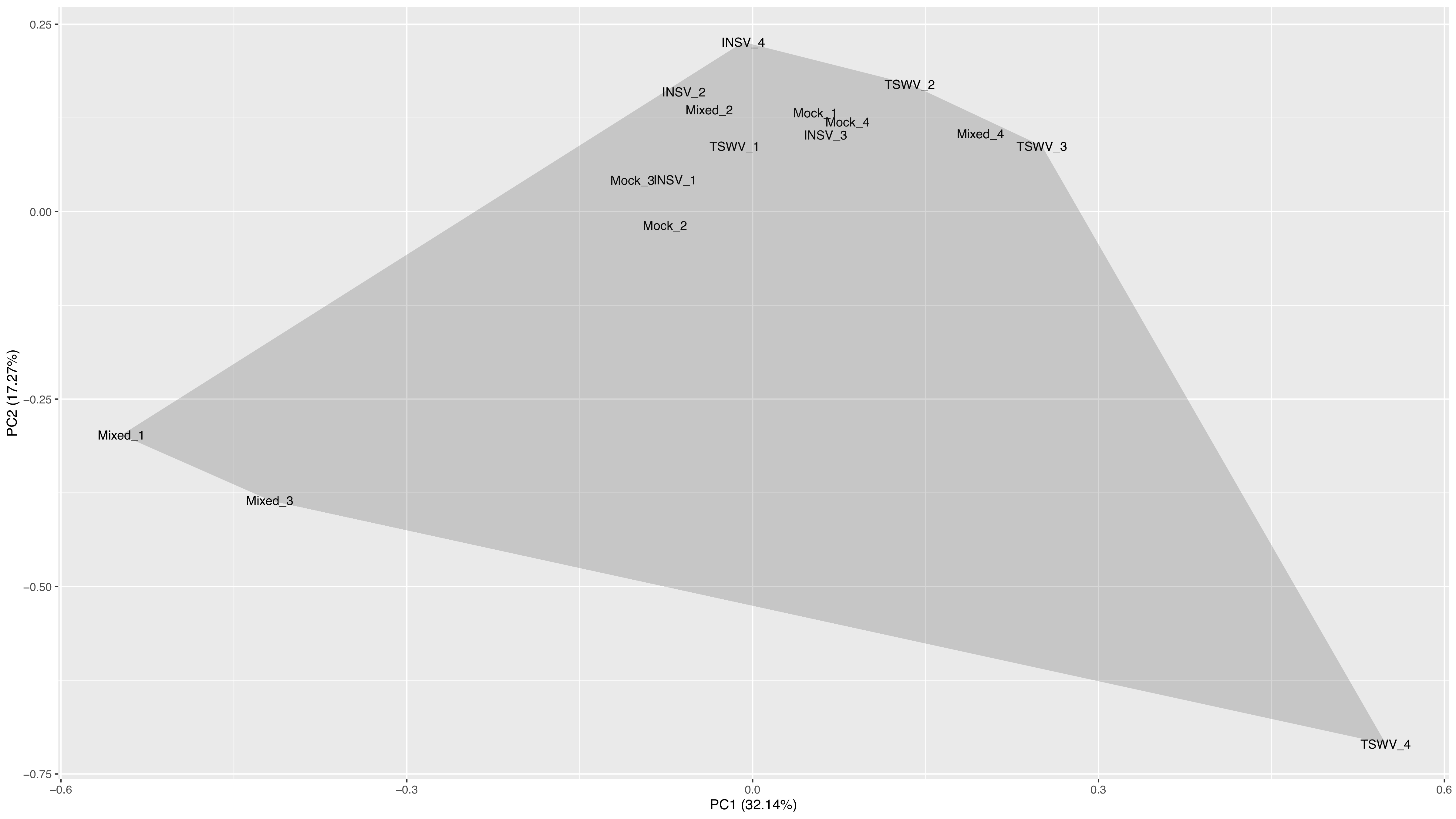

Supplement: Supplementary file 1 [file plants-09-00509-s001.zip › Supplementary Figure S1 PCA plot based on collected candidate volatile compounds .jpg]
